# Urinary 1-Hydroxypyrene is Associated with Oxidative Stress and Inflammatory Biomarkers in Acute Myocardial Infarction

**DOI:** 10.3390/ijerph110909024

**Published:** 2014-09-01

**Authors:** Fernando Freitas, Natália Brucker, Juliano Durgante, Guilherme Bubols, Rachel Bulcão, Angela Moro, Mariele Charão, Marília Baierle, Sabrina Nascimento, Bruna Gauer, Elisa Sauer, Marcelo Zimmer, Flávia Thiesen, Iran Castro, Paulo Saldiva, Solange C. Garcia

**Affiliations:** 1 Toxicology Laboratory, Department of Clinical and Toxicological Analysis, Federal University of Rio Grande do Sul, Porto Alegre, RS 90610-000, Brazil.; E-Mails: fernandofarmrs@yahoo.com.br (F.F.); nataliafarma@hotmail.com (N.B.); juliano_durgante@hotmail.com (J.D.); bubols@hotmail.com (G.B.); rachelbulcao@yahoo.com.br (R.B.); angelammoro@yahoo.com.br (A.M.); marifeiffercharao@yahoo.com.br (M.C.); mariliabaierle@yahoo.com.br (M.B.); sabrinascimento@hotmail.com (S.N.); bruna_gauer@hotmail.com (B.G.); elisa-sauer@hotmail.com (E.S.); 2 Department of Clinical and Toxicological Analysis, Federal University of Santa Maria, Santa Maria, RS 97105-900, Brazil; 3 Department of Clinical and Toxicological Analysis, University of Caxias do Sul, Caxias do Sul, RS 95070-560, Brazil; 4 Post-graduate Program of Pharmacy Sciences, Federal University of Rio Grande do Sul, Porto Alegre, RS 90610-000, Brazil; 5 Toxicology Institute, Pharmacy Faculty, Pontifical Catholic University of Rio Grande do Sul, Porto Alegre, RS 90619-900, Brazil; E-Mails: marcelo.zimmer@acad.pucrs.br (M.Z.); fvthiesen@pucrs.br (F.T.); 6 Institute of Cardiology, University Cardiology Foundation, Porto Alegre, RS 90620-000, Brazil; E-Mail: iran.castro@cardiologia.org.br; 7 Department of Pathology, College of Medicine, University of São Paulo, São Paulo, SP 05508-070, Brazil; E-Mail: pepino@usp.br

**Keywords:** 1-hydroxypyrene, lipid peroxidation, inflammation, biomarkers

## Abstract

Several studies have associated exposure to environmental pollutants, especially polycyclic aromatic hydrocarbons (PAHs), with the development of cardiovascular diseases. Considering that 1-hydroxypyrene (1-OHP) is the major biomarker of exposure to pyrenes, the purpose of this study was to evaluate the potential association between 1-OHP and oxidative stress/inflammatory biomarkers in patients who had suffered an acute myocardial infarction (AMI). After adopting the exclusion criteria, 58 post-infarction patients and 41 controls were sub-divided into smokers and non-smokers. Urinary 1-OHP, hematological and biochemical parameters, oxidative stress biomarkers (MDA, SOD, CAT, GPx and exogenous antioxidants) and the inflammatory biomarker (hs-CRP) were analyzed. 1-OHP levels were increased in post-infarct patients compared to controls (*p* < 0.05) and were correlated to MDA (*r* = 0.426, *p* < 0.01), CAT (*r* = 0.474, *p* < 0.001) and β-carotene (*r* = −0.309; *p* < 0.05) in non-smokers. Furthermore, post-infarction patients had elevated hs-CRP, MDA, CAT and GPx levels compared to controls for both smokers and non-smokers. Besides, β-carotene levels and SOD activity were decreased in post-infarction patients. In summary, our findings indicate that the exposure to pyrenes was associated to lipid damage and alterations of endogenous and exogenous antioxidants, demonstrating that PAHs contribute to oxidative stress and are associated to acute myocardial infarction.

## 1. Introduction

Environmental factors are considered important in the development of cardiovascular diseases, along with well-established risk factors such as lifestyle habits, including smoking, diet and exercise, which strongly influence the risk of developing coronary heart disease [1,2]. According to World Health Organization, the exposure to air pollution results in an estimated 7 million deaths each year. Recent epidemiological studies have shown that exposure to particulate matter (PM) in the environment is associated with increased cardiovascular mortality. Besides, a relationship was found between PM from the environment and heart ischemia, arrhythmias and stroke [3,4,5].

Emissions from vehicles contain a heterogeneous mixture of hazardous substances. The important fraction of air pollution in this context is particulate matter of less than 2.5 μm in size (PM_2.5_). These fine particles are released and derived from combustion processes, cigarette smoke, vehicles, industries or power plants [6]. Furthermore, PM_2.5_ contains different elements and toxic compounds such as toxic metals and polycyclic aromatic hydrocarbons (PAHs) adsorbed to its surface [7,8].

Toxicological evidence indicates that benzo[a]pyrene (BaP) is one of the most abundant PAHs found in the environment and is present in almost all mixtures of PAHs in relatively high concentrations (2%–10%) [9]. Humans are commonly exposed to PAH mixtures through the inhalation of polluted air and cigarette smoke [10]. Likewise, there are reports in the literature regarding the use of the urinary 1-hydroxypyrene (1-OHP) levels as biomarker to assess PAHs exposure [11,12,13].

Experimental studies indicate that the exposure to PAHs is associated with enhanced progression of atherosclerosis [14,15,16,17,18]. In order to investigate if and how the exposure to PAHs may contribute to the development of acute myocardial infarction (AMI) in humans, the objective of this study was to evaluate the potential associations among exposure to PAHs, more specifically pyrenes, and oxidative stress and inflammatory biomarkers in patients who had suffered an acute myocardial infarction.

## 2. Experimental Section

### 2.1. Subjects

One hundred and twenty-three patients (63.5 ± 9.6 years old) were admitted to the Institute of Cardiology of Rio Grande do Sul, in Porto Alegre, Brazil, after having suffered an acute myocardial infarction (AMI) and were included in this study. Subjects were diagnosed with AMI by their clinical symptoms, electrocardiogram and the following biochemical parameters: creatine kinase (CK) and creatine kinase MB fraction (CK-MB) enzymatic activities along with the determination of troponin levels.

Subjects who had some underlying diseases such as diabetes, cancer, kidney disease and pre-existing heart disease, or who had failed to provide urine samples during the blood collection, and also subjects who could not answer the questionnaire due to AMI complications were excluded from this study. The 58 patients remaining after adopting the exclusion criteria divided into two groups: post-infarction non-smoker subjects, aged 63.2 ± 9.1 years (*n* = 27), and post-infarction smoker subjects, aged 60.1 ± 10.2 years (*n* = 31). Regarding the control group, subjects were excluded from the study for the following criteria: history of ischemic heart disease, diabetes mellitus and chronic diseases, vitamin supplementation or subjects who failed to provide samples or participate in any stage of the study. A control group of 41 healthy subjects, aged 53.9 ± 7.8 years, was also divided into non-smokers, aged 55.7 ± 8.1 years (*n* = 25), and smokers, aged 52.2 ± 7.5 (*n* = 16).

All subjects answered an investigator-administered questionnaire to assess general health, lifestyle, smoking, alcohol drinking habits and use of medication, and also to determine the location of residence within the city. This study was approved by the Ethics Committee and Research from the Institute of Cardiology of Rio Grande do Sul (UP 4333/09) and all participants provided informed consent.

### 2.2. Collection of Biological Samples

In order to quantify 1-hydroxypyrene and creatinine, urine samples were collected from post-infarction patients, *i.e.*, patients after development of an AMI, as well as healthy controls. Venous blood samples were also collected from all patients immediately after the service at the hospital by venous puncture technique in Vacutainer^®^ tubes (BD Diagnostics, Plymouth, UK) containing sodium heparin, EDTA or without anticoagulants. Due to the immediate collection procedure, no fasting conditions could be achieved previously. Thus, the same criteria were performed to control group. Blood-EDTA was used for measurement to hemograms and carboxyhemoglobin levels, while plasma (obtained by centrifugation at 1500 g for 10 min at 4 °C) was used to determine malondialdehyde and non-enzymatic antioxidants. Blood-heparin was collected and stored at ‒80 ºC until analysis to determine endogenous enzymatic activities. Serum was used to determine lipid profile and high sensitivity C reactive protein.

### 2.3. Laboratorial Analyses

Hematological parameters were automatically obtained using a Sysmex XP 1800 system (Sysmex, Kobe, Japan). Fibrinogen was determined by a Sysmex CA-560 unit. A Cobas Integra 400 Plus^®^ (Roche Diagnostics, Basel, Switzerland) was utilized to analyze the serum inflammatory biomarker high sensitivity C reactive protein (hs-CRP) by the immunoturbidimetric method, and used to determine further biochemical parameters, such as total cholesterol, high density lipoprotein (HDL) cholesterol and triglycerides. Low density lipoprotein (LDL) cholesterol fraction was calculated by the Friedewald equation [19].

### 2.4. Quantification of Blood Carboxyhaemoglobin Levels

Carboxyhaemoglobin (COHb) was assayed in whole blood samples on the day of collection, according to the spectrophotometric method described by Beutler and West [20]. COHb levels were expressed as percentages.

### 2.5. 1-Hydroxypyrene Quantification

1-Hydroxypyrene (1-OHP), a biomarker of exposure to polycyclic aromatic hydrocarbons, was analyzed in urine samples. Briefly, urine (2.5 mL) was diluted with acetate buffer (5 mL, pH 5.0), then the enzyme β-glucuronidase (10 µL) was added to the samples, which were incubated at 37 ºC for 2 h. Subsequently, samples were submitted to solid phase extraction (SPE) procedure, in which 500 mg C18 cartridges (Chromabond^®^ MN, Macherey-Nagel, Düren, Germany) were equilibrated with high purity methanol (2 mL) and Milli-Q water (5 mL, Millipore, Bedford, MA, US). After loading the samples, cartridges were rinsed with 40% methanol (6 mL). Elution was carried out with isopropanol (2 mL) followed by evaporation under a stream of compressed air at 37 ºC and reconstitution with high purity methanol (200 µL) prior to injection into a high performance liquid chromatography (HPLC) system (Agilent^®^, Santa Clara, CA, USA) equipped with a 20 µL loop manual injector. Analyses were conducted under the following chromatographic conditions: a reverse-phase column (LiChospher check^®^ 100 RP-18) 150 mm × 4.6 mm with 5 µm particle size and a guard column (Eurospher-100) 5 mm × 4 mm with 5 µm particle size were utilized. The mobile phase was a mixture of Milli-Q water, acetonitrile and methanol (30:35:35, *v/v/v*). The flow rate was maintained isocratically at 1.0 mL/min, total run time was 12 min and a fluorescence detector was programmed to monitor excitation and emission of fluorescence at 242 nm and 388 nm wavelengths, respectively. The urinary 1-OHP levels were adjusted according to creatinine levels. Results were expressed as ng/g of creatinine. Urinary creatinine was measured by spectrophotometry, according to Jaffé, using commercial laboratory kits (Doles Reagents, Goiânia, GO, Brazil).

### 2.6. Lipid Peroxidation

Lipid peroxidation was determined by the measurement of malondialdehyde (MDA) levels. Plasmatic MDA was analyzed by high performance liquid chromatography with visible detection (HPLC-VIS), as described by Grotto *et al*. [21]. MDA levels are expressed as µmol/L.

### 2.7. Enzymatic Antioxidants

Catalase (CAT) activity was measured in 96-well microplates according to Aebi [22]. This assay is based on the decomposition of H_2_O_2_ by catalase. Enzymatic activity was evaluated by monitoring the rate of decrease in hydrogen peroxide (H_2_O_2_) absorbance at 240 nm during 5 min with readings every 20 s at 37 ºC. CAT activity was expressed as CAT units/mg of protein. The superoxide dismutase (SOD) activity was measured in 96-well microplates by the spectrophotometric method described by McCord and Fridovich [23], which is based on the inhibition of superoxide-dependent adrenaline auto-oxidation at 480 nm during 15 min with readings every 20 s at 32 ºC. SOD activity was expressed as SOD units/mg of protein. The enzymatic activity of glutathione peroxidase (GPx) was measured according to the method described by Paglia and Valentine [24] and absorbances were monitored at 340 nm during 6 min with readings every 20 s at 37 ºC. GPx activity was expressed as µmol of NADPH/min/mg of protein. CAT, SOD and GPx activities were quantified in a microplate reader (SpectraMax M2, Molecular Devices, Sunnyvale, CA, USA).

### 2.8. Non-Enzymatic Antioxidants

Simultaneous quantification of α-tocopherol, retinol, lycopene and β-carotene was performed according to a method developed in our laboratory [25] by high performance liquid chromatography (HPLC) with visible and fluorescence detection.

### 2.9. Statistical Analysis

Analysis of the data was performed using IBM SPSS Statistics software version 19 (IBM, Armonk, NY, USA). A normality test was applied to check the data distribution for each variable; variables that had non-normal distribution were log transformed. ANOVA followed by multiple comparison tests (Bonferroni *post hoc* test) was applied in the case of comparisons among more than two groups. Pearson rank correlations, log-transformed variables, were applied to evaluate correlation of 1-hydroxypyrene *vs.* different variables such as levels of MDA, β-carotene and CAT. In addition, multiple regression models were utilized to investigate whether 1-hydroxypyrene and other potential confounders (such as glucose, leukocytes, age and antioxidants exogenous) interfered with the with lipid peroxidation. Data are presented as mean ± standard error (SE). Statistical significance was considered when *p* < 0.05.

## 3. Results

**Figure 1:**
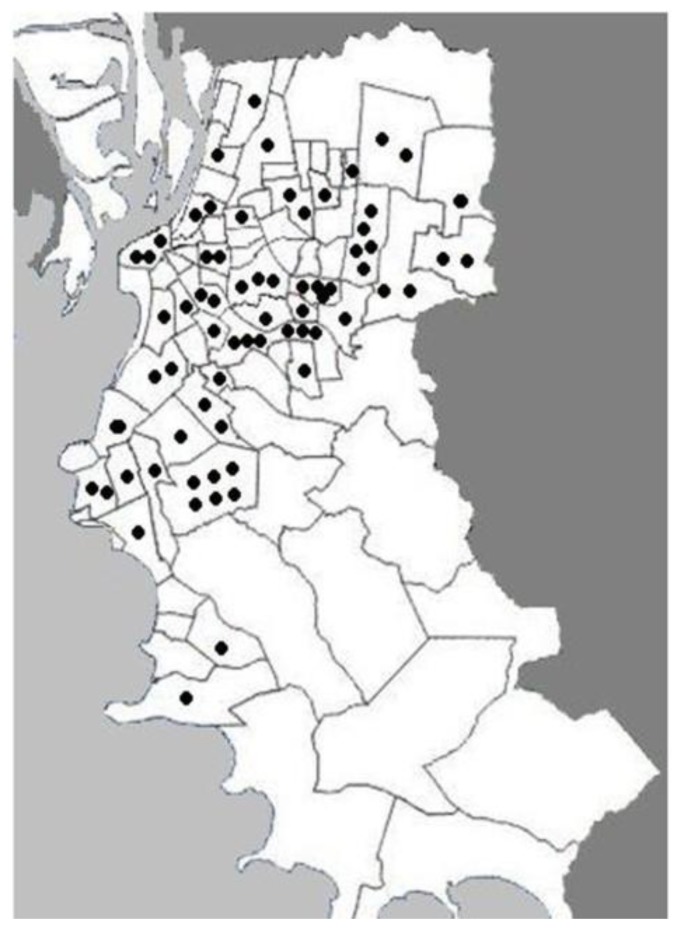
Geographic distribution of the households from the post-infarction patients enrolled in this study within the city of Porto Alegre, Brazil. The delimited areas represent neighborhoods and the dots indicate the location of each household in the neighborhoods.

Subjects who had suffered an AMI (post-infarction subjects) and controls were divided into smokers and non-smokers in order to exclude tabagism as a confounding factor. Baseline data of the population of study is presented in Table 1. No significant age differences were found among groups (*p* > 0.05). Post-infarction smokers presented a slightly higher sedentary lifestyle than post-infarction non-smokers. The patients enrolled in the study lived in different areas of the city; hence a homogeneous distribution was obtained (Figure 1).

**Table 1:** Baseline characteristics obtained from controls and subjects after an acute myocardial infarction.

Characteristics	Controls		Post-infarction	
	Non-Smokers (*n* = 25)	Smokers (*n* = 16)	Non-Smokers (*n* = 27)	Smokers (*n* = 31)
Age (years)	55.7 ± 8.1	52.2 ± 7.5	63.2 ± 9.1	60.1 ± 10.2
Gender (male/female)	(18/7)	(11/5)	(22/5)	(21/10)
Alcohol consumption (%)	38.5	6.5	40	45
Hypocholesterolemic drugs (%)	7.7	0	20.4	28.3
Anti hypertensive drugs (%)	30.8	3.5	43.4	40.2
Diuretic drugs (%)	4	0	18	22.4
Sedentary lifestyle (%)	8	58.8	55	70
Hypertension (%)	38.5	4.7	52	48

Note: Results are expressed as mean ± SE (standard error).

As shown in Table 2, several analyses were carried out in blood samples from the studied groups. Regarding the lipid profile and triglycerides, post-infarction subjects showed a significant reduction in comparison to controls, for both smoker and non-smokers (*p* < 0.05). No significant alteration of cholesterol fractions among the groups was observed (*p* > 0.05). It should be noted that the number of post-infarction patients undergoing treatment with hypocholesterolemic drugs such as statins in our study was higher than controls (Table 1) for both smokers and non-smokers, which could explain the significant decrease in their triglyceride levels.

**Table 2:** Hematological and biochemical analysis obtained from controls and subjects after an acute myocardial infarction.

Biomarkers	Controls		Post-Infarction	
	Non-Smokers (*n* = 25)	Smokers (*n* = 16)	Non-Smokers (*n* = 27)	Smokers (*n* = 31)
Platelets (10^3^/µL)	251 ± 10.7	243 ± 12.6	239 ± 8.4	259 ± 14.3
Fibrinogen (mg/dL)	270 ± 9.1	286 ± 20.9	250 ± 15.1	256 ± 13.8
Total cholesterol (mg/dL)	203 ± 8.8	238 ± 13.9 ^c^	207 ± 8.8	197 ± 7.4^b^
HDL cholesterol (mg/dL)	44.8 ± 2.8	44.6 ± 2.7	45.1 ± 2.8	41.1 ± 1.8
LDL cholesterol (mg/dL)	120 ± 14.4	154 ± 20.5	140 ± 17.3	135 ± 15.7
Total cholesterol /HDL	4.9 ± 0.3	5.7 ± 0.5	4.7 ± 0.3	5.0 ± 0.3
Triglycerides (mg/dL)	187 ± 27.8	197 ± 23.1	112 ± 13.5 ^a^	102 ± 8.4 ^b^

Notes: Results are expressed as mean ± SE (standard error). ^a^ *p* < 0.05 when compared to non-smokers controls; ^b^ *p* < 0.05 when compared to smokers controls; ^c^ *p* < 0.05 when compared to non-smokers controls (ANOVA/Bonferroni).

**Figure 2:**
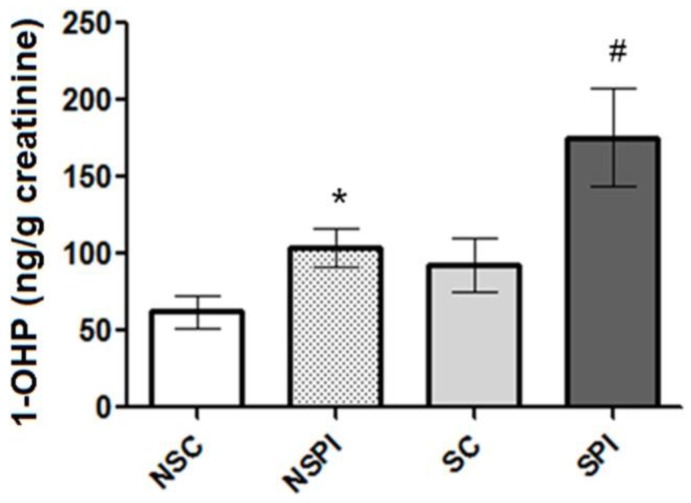
Urinary 1-hydroxypyrene (1-OHP) levels observed in the groups of study. Results are expressed as mean ± SE (standard error). NSC (non-smoker controls); NSPI (non-smoker post-infarction subjects); SC (smoker controls); SPI (smoker post-infarction subjects). * *p* < 0.01 for non-smokers (post-infarction compared to control). ^#^ *p* < 0.01 for smokers (post-infarction compared to control).

Carboxyhemoglobin (COHb) levels were analyzed for each group. However, no differences were found between COHb levels from post-infarction non-smokers *vs.* healthy non-smokers (*p* > 0.05) (data not shown). Figure 2 shows the results obtained from 1-OHP analyses carried out in this study. It is possible to observe that post-infarction subjects presented significant increase in urinary 1-OHP levels than controls (*p* < 0.01). Among post-infarction subjects, smokers (175.4 ± 31.94 ng/g creatinine) presented higher 1-OHP excretion than non-smokers (104.0 ± 12.87 ng/g creatinine) (*p* < 0.05). All analyses performed presented CV < 10%.

Table 3 depicts the results from the oxidative stress biomarkers analyzed for each group of study. Lipid damage biomarker, MDA, showed a significant increase in post-infarction subjects in comparison to controls, for both smokers and non-smokers (*p* < 0.05).

Furthermore, we analyzed enzymatic antioxidant system in all groups (Table 3). A decreased superoxide dismutase (SOD) activity was found in post-infarction subjects *vs.* controls, for both smokers and non-smokers (*p* < 0.05). On the other hand, we observed an increase in catalase (CAT) and glutathione peroxidase (GPx) activities in post-infarction subjects in comparison to controls, for smokers and non-smokers (*p* < 0.05). Non-enzymatic antioxidants have also been evaluated, as shown in Table 3. Also, a decrease in β-carotene content in post-infarction subjects *vs.* controls has been found in both smokers and non-smokers (*p* < 0.05). No significant changes were found for α-tocopherol, retinol and lycopene levels (*p* > 0.05) among groups.

**Table 3:** Biomarkers of oxidative stress obtained from controls and subjects after an acute myocardial infarction.

Biomarkers	Controls		Post-infarction	
	Non-Smokers (*n* = 25)	Smokers (*n* = 16)	Non-Smokers (*n* = 27)	Smokers (*n* = 31)
MDA (µmol/L)	6.1 ± 0.3	7.6 ± 0.7	11.7 ± 0.8 ^a^	11.7 ± 0.9 ^b^
SOD (U/mg protein)	13.8 ± 1.2	21.5 ± 1.9 ^c^	10.3 ± 1.1 ^a^	12.7 ± 1.2 ^b^
CAT (U/mg protein)	12.1 ± 1.0	10.5 ± 1.0	30.2 ± 1.8 ^a^	30.2 ± 2.1 ^b^
GPx (µmol NADPH/min/mg protein)	7.5 ± 0.5	8.4 ± 0.8	11.3 ± 0.7 ^a^	12.0 ± 0.7 ^b^
Vitamin E (µmol/L)	29.6 ± 2.0	34.0 ± 2.2	31.4 ± 2.7	30.1 ± 2.4
Retinol (µmol/L)	2.3 ± 0.1	2.2 ± 0.1	2.7 ± 0.1	2.4 ± 0.1
β-Carotene (µmol/L)	0.8 ± 0.08	0.74 ± 0.07	0.4 ± 0.1 ^a^	0.4 ± 0.1 ^b^
Lycopene (µmol/L)	0.6 ± 0.1	0.5 ± 0.1	0.7 ± 0.1	0.7 ± 0.1

Notes: Results are expressed as mean ± SE (standard error). ^a^ *p* < 0.05 when compared to non-smokers controls; ^b^ *p* < 0.05 when compared to smokers controls; ^c^ *p* < 0.05 when compared to non-smokers controls (ANOVA/Bonferroni).

High sensitivity C reactive protein (hs-CRP), an important inflammation biomarker, was found to be significantly increased in post-infarction subjects in relation to controls, for both smokers (0.87 ± 0.2 *vs.* 0.39 ± 0.05, *p* < 0.05) and non-smokers (0.86 ± 0.28 *vs.* 0.3 ± 0.05, *p* < 0.05).

**Figure 3:**
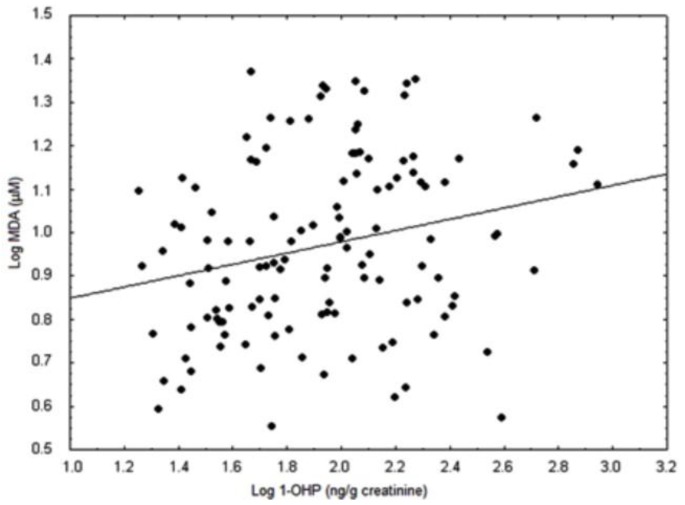
Association of plasmatic malondialdehyde (MDA) *vs.* urinary 1-hydroxypyrene (1-OHP) levels in non-smoker subjects, (*r* = 0.426; *p* = 0.01; *n* = 52).

The lipid damage was positively correlated with 1-OHP urinary excretion (*r* = 0.426; *p* < 0.01; Figure 3). Furthermore, 1-OHP urinary showed positive correlation with CAT activity (*r* = 0.474, *p* < 0.001) and negative correlation with β-carotene (*r* = −0.309, *p* < 0.05). Multivariate regression models were adopted to evaluate the increase in MDA levels. The best fit multivariate regression model accounted for 57% of the increased MDA levels, and among the independent variables analyzed, urinary 1-OHP explains the lipid peroxidation (β estimate = 0.170, *p* = 0.017).

## 4. Discussion

Epidemiological studies have reported that PAH exposure has adverse effects on human health, including the cardiovascular system [26]. To our knowledge there are no studies investigating the additional influence of exposure to PAHs in AMI, through the evaluation and comparison of the biomarkers of exposure and effect in patients after an acute myocardial infarction and controls. Studying the possible association of urinary 1-hydroxypyrene with biomarkers of effect such as oxidative stress and inflammatory biomarkers becomes relevant due to the involvement of these biomarkers in the atherosclerotic process, which may lead to the development of AMI.

Previous studies have suggested that the quantification of urinary 1-OHP can be used as a representative biomarker for assessing PAHs exposure present in environmental pollution [13,27,28,29]. However, the excretion of 1-OHP in urine can be influenced by lifestyle factors, such as food intake. In our study, increase in the urinary 1-OHP level was found for both smokers and non-smokers post-infarction compared to the controls. As a matter of fact, it has been observed that smokers presented higher values of 1-OHP excretion than non-smokers, indicating that smokers present an additional source of PAHs exposure, i.e. cigarette smokes [30]. On the other hand, blood COHb did not demonstrate enough specificity to be adopted as a biomarker of environmental exposure [31], considering the lack of significant difference between controls and AMI patients, but only between smokers and non-smokers.

Table 3 depicts the results from the oxidative stress biomarkers analyzed for each group of study. Regarding the lipid damage biomarker, post-infarction subjects showed a significant increase of MDA levels in comparison to controls, for both smokers and non-smokers. Our results are in agreement with another study from Bagatini *et al.* [32], which suggested that post-infarction patients had increased lipid oxidation. In contrast to other works, this study showed that the MDA levels were positively associated with 1-OHP, which demonstrated that high levels of PAHs exposure contribute to the increase lipid oxidation.

Our results also demonstrate a decrease in superoxide dismutase (SOD) activity in post-infarction patients. SOD is considered a key enzyme in the depletion of superoxide radicals (O_2_·^−^), which are considered primary ROS once they are known to culminate in the formation of further reactive species, the so-called secondary ROS, such as hydroxyl radicals (·OH) and hydrogen peroxide (H_2_O_2_) [33,34]. Once SOD is activated, it is known to increase the formation of H_2_O_2_ as a result of the enzymatic activity in an attempt to lower O_2_·^−^ levels. In this study, the decrease in SOD activity could be a consequence of a prior enzymatic activation, which was responsible to diminish O_2_·^−^ levels, thus resulting in the formation of H_2_O_2_. In this case, it is likely that SOD activation could not be detected once a decrease in enzymatic activity had already been stimulated by superoxide anion decrease. On the other hand, we found an activation of both CAT and GPx enzymes which could be connected as an attempt to scavenge and inactivate secondary ROS, such as hydrogen peroxide, by a compensatory antioxidant system [35,36].

Some studies have suggested that carotenoids scavenge peroxyl radicals by inflow of hydrogen and addition or electron transfer [37]. In this context, β-carotene is an important non-enzymatic exogenous antioxidant that can act by scavenging free radicals and reactive oxygen species before they exert their deleterious effects [38]. We found that β-carotene levels were diminished in patients with acute myocardial infarction compared with controls, for both smokers and non-smokers.

Moreover, CAT activity and β-carotene levels showed correlations with 1-OHP levels, which suggest that exposure to PAHs may be responsible for the alterations of endogenous and exogenous antioxidants.

Serum hs-CRP levels were increased in post-infarction patients, indicating that this inflammatory biomarker is linked to the acute myocardial infarction. Despite the increase in this inflammatory biomarker, no correlation of hs-CRP with excretion of the biomarker of exposure, 1-OHP, was found. In this scenario, the involvement of additional inflammatory cytokines and adhesion molecules could be investigated and possibly correlated with 1-OHP excretion.

The environmental exposure to PAHs observed by an increase of 1-OHP, and its correlations with important oxidative stress biomarkers such as MDA, enzymatic antioxidant system (catalase) and the non-enzymatic antioxidant β-carotene, are reported in this study. According to the multiple linear regression analyses performed in this work, the urinary 1-OHP levels contribute for the lipid peroxidation. Therefore, as far as we know, this is the first study that associates the urinary biomarker of exposure to pyrenes, 1-OHP, in AMI patients and lipid peroxidation, as well as actions on antioxidant compounds, such as carotenoids and catalase, suggesting a potential contribution of PAH to lipid damage in IAM subjects. However, additional studies are necessary.

The present study presents some limitations such as the small number of subjects studied, which might limit the generalization of our results, and the influence that other urban pollutants may have on the markers of oxidative stress. Moreover, the present work only may suggest association between 1-OHP in urine and lipid damage in AMI patients as a pilot study, and more studies are required to determine the causality of these associations.

## 5. Conclusions

Taken together, the present results indicated that the exposure to environmental PAHs could be suggested as contributing to development of AMI in non-smoker subjects, probably through an induction of oxidative stress, depletion of exogenous antioxidant levels, and also by an increase in the pro-inflammatory biomarker hs-CRP. However, further investigations are necessary to confirm this suggestion. In this line, more inflammatory biomarkers and preferably studies adopting *in vivo* models will be necessary to first to confirm and second understand how the activation of inflammation is influenced by the exposure to PAHs and the onset of acute myocardial infarction.
